# Methodological reporting of randomized controlled trials in major hepato-gastroenterology journals in 2008 and 1998: a comparative study

**DOI:** 10.1186/1471-2288-11-110

**Published:** 2011-07-30

**Authors:** Ji-Lin Wang, Tian-Tian Sun, Yan-Wei Lin, Rong Lu, Jing-Yuan Fang

**Affiliations:** 1 Department of Gastroenterology, Shanghai Jiao-Tong University School of Medicine Ren-Ji Hospital, Shanghai Institute of Digestive Disease. 145 Middle Shandong Road, Shanghai, 200001, China

## Abstract

**Background:**

It was still unclear whether the methodological reporting quality of randomized controlled trials (RCTs) in major hepato-gastroenterology journals improved after the Consolidated Standards of Reporting Trials (CONSORT) Statement was revised in 2001.

**Methods:**

RCTs in five major hepato-gastroenterology journals published in 1998 or 2008 were retrieved from MEDLINE using a high sensitivity search method and their reporting quality of methodological details were evaluated based on the CONSORT Statement and Cochrane Handbook for Systematic Reviews of interventions. Changes of the methodological reporting quality between 2008 and 1998 were calculated by risk ratios with 95% confidence intervals.

**Results:**

A total of 107 RCTs published in 2008 and 99 RCTs published in 1998 were found. Compared to those in 1998, the proportion of RCTs that reported sequence generation (RR, 5.70; 95%CI 3.11-10.42), allocation concealment (RR, 4.08; 95%CI 2.25-7.39), sample size calculation (RR, 3.83; 95%CI 2.10-6.98), incomplete outecome data addressed (RR, 1.81; 95%CI, 1.03-3.17), intention-to-treat analyses (RR, 3.04; 95%CI 1.72-5.39) increased in 2008. Blinding and intent-to-treat analysis were reported better in multi-center trials than in single-center trials. The reporting of allocation concealment and blinding were better in industry-sponsored trials than in public-funded trials. Compared with historical studies, the methodological reporting quality improved with time.

**Conclusion:**

Although the reporting of several important methodological aspects improved in 2008 compared with those published in 1998, which may indicate the researchers had increased awareness of and compliance with the revised CONSORT statement, some items were still reported badly. There is much room for future improvement.

## Background

In the field of evidence-based medicine, randomized controlled trials (RCTs) with a logical design and correct implementation are considered to provide the best evidence for healthcare interventions [1]. In order to assess RCTs accurately, readers need complete, clear, and transparent information with regards to the design and conduction of the trials. Inappropriate experimental design and/or reporting usually lead to confusion in data interpretation. For example, unclear or inadequate allocation concealment is associated with an overestimation of treatment effect [2-4]. Therefore, adequate reporting of clinical trials, especially the methodological characteristics, is crucial for readers to appraise their validity.

In order to improve the quality of reporting in RCTs, the Consolidated Standards of Reporting Trials (CONSORT) statement was developed. Since it was published in 1996, it has been gradually accepted by many medical journals and has been associated with improvement of the quality of RCT reporting [5-8]. The statement was subsequently revised in 2001 and updated in 2010. We conducted this study in an attempt to evaluate the reporting qualities of key methodological items in RCTs from five major hepato-gastroenterology journals in 1998, 3 years before the revised version of CONSORT, and in 2008, 7 years after its revised version. We assessed whether the methodological reporting quality has improved over this10 year period, especially before and after the publication of the revised version of the CONSORT in 2001.

## Methods

### Information source

We used a highly sensitive search strategy based on the Cochrane Handbook for Systematic Reviews of Interventions (Version 5.0.2) [9] to retrieve relevant RCTs published in the five highest impact factor journals of gastroenterology and hepatology (American Journal of Gastroenterology, Gastroenterology, Gut, Hepatology, and the Journal of Hepatology) in 1998 or 2008 from MEDLINE. In order to identify missing RCTs, these five journals were also manually searched.

### Inclusion criteria

Only RCTs were eligible in our analysis. The trial was defined as a RCT if "random" was mentioned when participants were assigned to interventions. The following trial reports were excluded: reports published in a summary form or as reviews of randomized trials, reports on randomized trials on animals or healthy volunteers, and reports that did not describe the intervention outcome of randomly allocated patients.

### Data extraction

Two authors (JW & TS), who were blinded to each other's results, screened all titles and abstracts of possibly relevant RCTs, and then selected RCTs in accordance with the predetermined eligibility criteria; studies were discarded that were not applicable by reviewing the full texts. One author (JW) extracted data from all articles, while another (TS) randomly selected 50 RCTs (24% of the sample) and extracted data independently using the same methods. Differences generated in data extraction were resolved by discussion and the senior reviewer (JF) was asked for help when disagreement could not be resolved by discussion.

### Standards of methodological reporting

Based on the Cochrane Handbook for Systematic Reviews of Interventions and the CONSORT statement [10], seven items that were deemed important to avoid bias of the effect estimation were extracted for assessment. The seven methodological items were as follows: (1) random allocation sequence generation, classified as adequate reporting (when methods used to generate random sequence, randomization type, and restriction details were all reported), partial reporting (only parts of detials were reported), and no reporting; (2) allocation concealment, classified as adequate reporting (when mechanism used to implement random sequence, and whether concealed sequence until interventions assigned were both reported), partial reporting (only parts of details were reported), and no reporting; (3) blinding, classified as adequate reporting (people who were blinded after assignment to interventions and how blinding was conducted, description of the similarity of interventions were all reported), partial reporting (only parts of details were reported), and no reporting; (4) sample size calculation, classified as adequate reporting (description of the methods of determining sample size and explanation of any interim analyses and stopping guidelines), partial reporting (only parts of details were reported), and no reporting; (5) incomplete outcome data addressed, classified as adequate reporting (missing outcome data was shown and whether the missing outcome data had a clinically relevant impact on intervention effect estimate), partial reporting (only parts of details were reported), and no reporting; (6) intention to treat analysis, classified as adequate reporting (how many participants were included in each analysis and whether the analysis was by original assigned groups), partial reporting (only parts of details were reported), and no reporting; (7) free of selective reporting, classified as adequate reporting(reporting all planned primary and secondary end points), partial reporting (only parts of details were reported), and no reporting.

### Data analysis

Cohen's kappa analysis [11] was performed to measure the level of agreement between the reviewers on all items of the data abstraction form. Agreement was judged as poor (kappa < 0.2), fair (0.21< kappa < 0.4), moderate (0.41< kappa < 0.6), substantial (0.61< kappa < 0.8), or good (kappa > 0.8).

We reported categorical data as frequencies, percentages, and 95% confidence interval (95%CI), using the Wilson Scoring method. Differences of the reporting quality of key methodological items in proportions in 1998 versus 2008 were tested using Chi-square analysis, and expressed as risk ratio (RR) with 95%CI. P-values were two-tailed and those < 0.05 were considered statistically significant. All of the statistical analysis was conducted using SAS 9.2 software (SAS Institute Inc. USA).

## Results

### Search results

**Figure 1:**
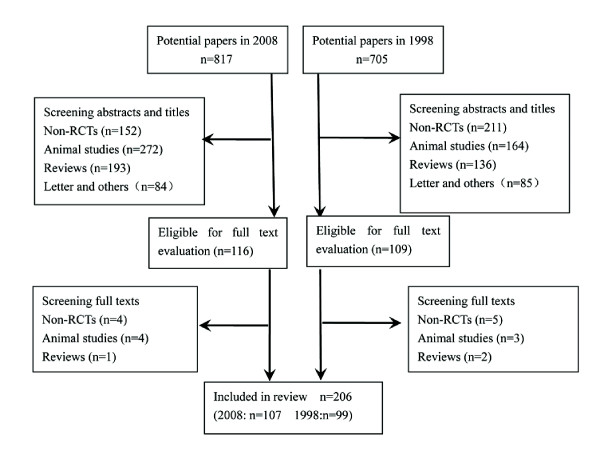
**Flow diagram of the search strategy and review process**.

Flow diagram of the search strategy and review process was detailed in Figure 1. Finally, a total of 206 RCTs (107 in 2008 and 99 published in 1998) were included. Kappa scores of data extracted independently by the two authors (JW and TS) were all greater than 0.80, indicating good agreement.

### Characteristics of included trials

RCTs published in 2008 with a median of 167 participants and a median of 356 days follow-up period covered 26 diseases, while RCTs in 1998, with a median of 94 participants and a median of 178 days follow-up period, covered 24 diseases. In 1998, single-center trials were more common than multi-center trials, but in 2008 more than 70% of trials were conducted in multiple centers. Funding source information is important for readers to assess therapeutic effects. The number funded by industry increased in 2008. Characteristics of the included trials were detailed in Table 1.

**Table 1:** Characteristics of included RCTs

Administrative indicators	Trials in 2008	Trials in 1998	RR [95%CI]	p value
			
	No. (%)	No. (%)		
**Top five kinds of disease**				

chronic hepatitis C	19(18%)	15(15%)	1.21 [0.58, 2.53]	P = 0.62

inflammatory bowel disease	17(16%)	11(11%)	1.51 [0.67, 3.41]	P = 0.32

liver cirrhosis	11(10%)	12(12%)	-	-

chronic hepatitis B	12(11%)	-	-	-

colorectal cancer	8(7%)	-	-	-

peptic ulcer	-	15(15%)	-	-

liver cancer	-	5(5%)	-	-

**Region**				

North America	36(34%)	30(30%)	1.17 [0.65, 2.10]	P = 0.61

Europe	42(39%)	56(57%)	0.50 [0.28, 0.86]	P = 0.01

Asia	19(18%)	8(8%)	2.46 [1.02, 5.90]	P = 0.04

Australia	10(9%)	4(4%)	2.45 [0.74, 8.08]	P = 0.14

**Center**				

Single-center	31(29%)	51(52%)	0.38 [0.22, 0.68]	P = 0.001

Multi-center	76(71%)	48(48%)	2.60 [1.47, 4.63]	P = 0.001

**Funding source**				

Industry	43(40%)	25(25%)	1.99 [1.10, 3.61]	P = 0.02

Public	40(38%)	32 (32%)	1.25 [0.70, 2.22]	P = 0.45

Public and industry	13(12%)	8(8%)	1.57 [0.62, 3.97]	P = 0.34

Not specified	5(5%)	33(33%)	0.10 [0.04, 0.26]	P < 0.001

None	6(5%)	1(1%)	5.82 [0.69, 49.24]	P = 0.11

**Ethics committee approval**	105(98%)	86(87%)	7.94 [1.74, 36.13]	P = 0.007

**Informed consent from patients**	100(93%)	81(82%)	3.17 [1.26, 7.97]	P = 0.01

### Reporting of methodological items

Referring only to the items reported adequately, there was an increase in the proportion of trial reports that included adequate details of sequence generation (RR, 2.44; 95%CI 1.09-5.42; 10% in 1998 vs. 22% in 2008), sample size calculation (RR, 2.34; 95%CI 1.14-4.84; 13% in 1998 vs. 28% in 2008), intention-to-treat analysis (RR, 1.94; 95%CI 1.1-3.45; 30% in 1998 vs. 46% in 2008) between 1998 and 2008. Considering items reported adequately or partly as they had been reported, RCTs in 2008 had better reporting of the methodological items of sequence generation (RR, 5.70; 95%CI 3.11-10.42; 35% in 1998 vs. 76% in 2008), allocation concealment (RR, 4.08; 95%CI 2.25-7.39; 25% in 1998 vs. 62% in 2008), sample size calculation (RR, 3.83; 95%CI 2.10-6.98; 47% in 1998 vs. 78% in 2008), intention-to-treat analyses (RR, 3.04; 95%CI 1.72-5.39; 42% in 1998 vs. 69% in 2008), incomplete outcome data addressed (RR, 1.81; 95%CI 1.03-3.17; 34% in 1998 vs. 49% in 2008) than those published in 1998. However, no clear difference emerged in the reporting of blinding (RR, 1.25; 95%CI 0.72-2.16), free of selective reporting (RR, 1.48; 95%CI 0.86-2.57), and free of other bias (RR, 0.90; 95%CI 0.50-1.61) between the two selected years (Table 2).

**Table 2:** Reporting quality of key methodological items

Items	2008(n = 107)	1998(n = 99)	2008 VS 1998	
	
	n(%)	n(%)	RR [95%CI]	*P *value
**Sequence generation**				

adequate reporting	23(22%)	10(10%)	2.44[1.09,5.42]	< 0.05

partial reporting	58(54%)	25(25%)		< 0.05

no reporting	26(24%)	64(75%)		< 0.05

*adequate + partial reporting	81(76%)	35(35%)	5.70[3.11,10.42]	< 0.05

**Allocation concealment**				

adequate reporting	6(6%)	3(3%)	1.90[0.46,7.82]	0.89

partial reporting	56(52%)	22(22%)		< 0.05

no reporting	45(42%)	74(75%)		< 0.05

*adequate + partial reporting	62(58%)	25(25%)	4.08 [2.25, 7.39]	< 0.05

**Blinding**				

adequate reporting	24(22%)	22(22%)	1.01[0.52,1.95]	0.97

partial reporting	38(36%)	30(30%)		0.43

no reporting	45(42%)	47(47%)		0.43

*adequate + partial reporting	62(58%)	52(53%)	1.25 [0.72, 2.16]	0.43

**Sample size calculation**				

adequate reporting	28(26%)	13(13%)	2.34[1.14,4.84]	< 0.05

partial reporting	55(52%)	34(34%)		< 0.05

no reporting	24(22%)	52(53%)		< 0.05

*adequate + partial reporting	83(78%)	47(47%)	3.83 [2.10, 6.98]	< 0.05

**Incomplete outecome data addressed**				

adequate reporting	20(19%)	12(12%)	1.67[0.77,3.62]	0.2

partial reporting	32(30%)	22(22%)		0.21

no reporting	55(51%)	65(66%)		< 0.05

*adequate + partial reporting	52(49%)	34(34%)	1.81[1.03,3.17]	< 0.05

**Intention-to-treat analysis**				

adequate reporting	49(46%)	30(30%)	1.94[1.10,3.45]	< 0.05

partial reporting	25(23%)	12(12%)		< 0.05

no reporting	33(31%)	57(58%)		< 0.05

*adequate + partial reporting	74(69%)	42(42%)	3.04 [1.72, 5.39]	< 0.05

**Free of selective reporting**				

adequate reporting	34(32%)	20(20%)	1.84[0.97,3.48]	0.06

partial reporting	23(21%)	23(23%)		0.76

no reporting	50(47%)	56(57%)		0.16

*adequate + partial reporting	57(53%)	43(43%)	1.48[0.86,2.57]	0.16

### Methodological reporting quality in 2008 according to different strata

We explored the association between the methodological reporting quality and center and funding source. In this section, we also took the items reported adequately or partly as they had been reported. The results showed that the methods of blinding and intention-to-treat analysis are related to the number of centers involved. Multi-center trials had better reporting of these two methodological items than single-center trials. As for the effects of different funding, allocation concealment and blinding were observed to be reported better in industry-sponsored trials than in public-funded trials. The reporting of other items were not associated with center or funding source (Table 3).

**Table 3:** Methodological reporting in 2008 according to center and funding source

	Sequence generation	Allocation concealment	Blinding	Sample size calculation	Incomplete outecome	Intention-to-treat analysis	Free of selective reporting
	
	n (%)	n (%)	n (%)	n (%)	n (%)	n (%)	n (%)
**Center**							

Single-center	20(64%)	14(45%)	13(42%)	21(68%)	17(55%)	17(55%)	21(68%)
(n = 31)							
	
Multi-center	61(80%)	48(63%)	49(64%)	62(82%)	35(46%)	57(75%)	36(47%)
(n = 76)							
	
P value	0.08	0.09	0.03	0.12	0.41	0.04	0.06

**Funding source**							

Industry	37(86%)	31(72%)	30(70%)	34(80%)	18(42%)	28(65%)	21(49%)
(n = 43)							

Public	28(70%)	20(50%)	19(48%)	30(75%)	23(58%)	28(72%)	25(63%)
(n = 40)							

P value	0.07	0.04	0.04	0.66	0.16	0.64	0.21

## Discussion

Accurate description of methodology is essential for readers to assess the internal and external validity of RCTs. In this study, reporting quality of key methodological items of RCTs published in five major gastroenterology and hepatology journals in 2008 or 1998 were systematically assessed and compared. We identified an improvement in the key items of sequence generation, allocation concealment, sample size calculation, intention-to-treat analyses, and addressing of incomplete outcome data in 2008 compared with 1998. Nevertheless, the methodological reporting quality was still unsatisfactory.

Although the method of sequence generation was reported in more than twice as many articles published in 2008 than in 1998, 24% of articles still had inadequate information about this item. According to the latest CONSORT statement [10], adequate description of random allocation sequence generation should contain at least three aspects: methods used to generate the random sequence, randomization type, and restriction details. Applying these criteria to assessment of these articles, the results were even more unsatisfactory. In 2008, 12 years after the CONSORT statement was first published, only 23 articles adequately described how the random allocation sequence was generated. Sequence concealment allocation is also crucial to avoid bias, but this item was not reported well either. In 1998, only a quarter of articles reported that generated allocation schedules were implemented by allocation concealment. Ten years later, only half of trials reported adopting allocation concealment. Without adequate information, readers cannot define the exact method used. At the same time, without adequate information readers were unclear whether the randomization process was free from human alteration and whether the findings were valid because trials with poor or unexplained concealment were more likely to yield larger estimates of treatment effect [12]. Therefore, not only details of the mechanism used to implement the random allocation sequence are required, but also the steps taken to conceal the sequence, and who generated the random allocation sequence, enrolled and assigned participants are required.

In 1998, blinding was the best reported item among the seven key methodological items, but no significant improvement was observed in the next 10 years. This finding was in agreement with Amy's conclusion that CONSORT adoption had little effect on blinding of participants [13]. According to the CONSORT statement, people who were blinded after assignment to interventions and how the blinding was conducted should be all reported. However, many articles only described trials as "double blinded" or "blinded", without providing any details. In trials without reporting of blinding details, bias may occur either intentionally or unintentionally, so their results are not fully reliable. For patient-reported outcomes in particular, result surveyors should make every effort to eliminate measurement bias. A meta-epidemiological study found that effect estimates were exaggerated when blinding had not taken place in trials with subjective outcomes effect estimate [14].

An adequate number of participants is essential for the detection of clinically significant differences with a high power. From the results of previous studies [15-18] and our article, the reporting quality of this item is gradually improving. The internal validity of RCTs is also associated with study participation and continuation. Omitting participants withdrawing from the trials easily reintroduced imbalance and prevented readers from calculating the attrition rates for different experimental conditions, which led to an overestimate of treatment effectiveness [19]. However, this methodological item was seldom assessed in previous articles evaluating the methodological reporting of RCTs about digestive disease. To our knowledge, our study and Kjaergard's study [16] are the only two studies evaluating the reporting condition of this item. Kjaergard only examined RCTs published in Hepatology and found that drop-outs and withdrawals were adequately described in 70% of RCTs. However, this item was reported only in 30% of RCTs published in the five major gastroenterology and hepatology journals in 1998 and 49% of RCTs published in 2008.

As for the effects of funds upon methodological reporting quality, though there were different opinions [20,21], industry-sponsored trials were reported to have better reporting of methodology than non-industry sponsored trials [15,22,23]. For example, both Brown A [22] and Thomas O's [23] study have found that trials with industry funding had a higher methodology score than those with public funding. Bai Y's study [15] indicated that those industry-sponsored trials had better reporting of double blinding than public sponsored trials. We also found that industry-sponsored trials were significantly more likely to report the item of sequence allocation concealment and blinding. Compared with single-center trials, more participants were recruited by the multi-center collaboration, which resulted in an increase in test performance [24]. We examined the difference of methodological reporting quality between single-center and multi-center trials and found only blinding and "intention to treatment " analysis were better in multi-center trials though it was believed that prospective studies, undertaken in collaboration (either jointly or in parallel), could lead to the development of treatments that are truly beneficial for patients with these diseases [25].

In addition, we also compared this study with previous studies [15-18] focusing on reporting methodological quality of RCTs published in one or more gastroenterology and hepatology journals, and found an improvement of almost all methodological items after the revised version of CONSORT in 2001(Shown in Table 4). Compared with those studies [15-18], we have done a more comprehensive evaluation of the methodological reporting, while some of the methodological items were seldom assessed in previous studies, especially the items related to incomplete outcome, ITT analysis and free of selective reporting, which are also important for readers to assess the internal validity of the RCTs.

**Table 4:** Methodological reporting in major hepato-gastroenterology journals in different years

	**Gluud **[17]	**Kjaergard **[16]	Current study	**Kjaergard **[18]	**Bai **[15]	Current study
**Study period**	1985-1997	1981-1998	1998	1964-2000	2006	2008

**Included journals**	the Journal of Hepatology	Hepatology	5 *	Gastroenterology	6*	5*

**Number of RCTs**	166	235	99	383	105	107

**Sequence generation**	47(28%)	121(51%)	35(35%)	161(42%)	85(81%)	81(76%)

**Allocation concealment**	22(13%)	80(34%)	25({25%)	149(39%)	64(61%)	62(58%)

**Blinding**	50(30%)	80(34%)	52(53%)	237(62%)	54(51%)	62(58%)

**Sample size calculation**	33(20%)	61(26%)	47(47%)	-	79(75%)	83(78%)

**Incomplete outcome**	-	165(70%)	34(34%)	-	-	52(49%)

**Intention-to-treat analysis**	95(57%)	-	42(42%)	-	-	74(69%)

**Free of selective reporting**	-	-	43(43%)	-	-	57(53%)

*The current study included the five journals:American Journal of Gastroenterology, Gut, the Journal of Hepatology, Gastroenterology, Hepatology.

* The study of Bai included six journals:Gastroenterology, Hepatology, Gut, Journal of Hepatology, American Journal of Gastroenterology,

Clinical Gastroenterology and Hepatology

In this study, we used a standardized and rigorous evaluation instrument to assess the reporting of key methodological items systematically, and the abstraction processes were independently performed by two qualified reviewers. However, there were still limitations in this study. Firstly, we only included five major journals and only assessed RCTs for two years due to time and resource constraints, so the results could not represent the methodological reporting quality of the entire range of gastroenterology and hepatology journals. Secondly, we did not compare the methodological reporting quality of these five journals before and after adopting the CONSORT statement. We did attempt to find out when the CONSORT statement was adopted by each journal, but were unable to do so for three of them.

## Conclusions

Our analysis, although not exhaustive, suggests a significant improvement between 1998 and 2008 in the reporting quality of key methodological items in the major gastroenterology and hepatology journals, which is most likely the result of compliance with the CONSORT Statement. However, we can see that there is still ample room for improvement. Now the CONSORT Statement 2010 edition has been published, we call on authors and journal editors, especially those in Asia, to support and implement it in order to enhance the quality of RCT reporting.

## Competing interests

The authors declare that they have no competing interests.

## Authors' contributions

JW and TS designed this study and extracted the data. JW, TS and YL conducted the statistical analyses and created the first draft of the manuscript. All authors participated in editing the manuscript and approved final manuscript for publication.

## Funding

This work was supported by a grant from the Ministry of Public Health, China (No: 200802094) and the National Science Found of China (30830055) for Fang JY.

## Pre-publication history

The pre-publication history for this paper can be accessed here:

http://www.biomedcentral.com/1471-2288/11/110/prepub
